# Bogazici mouse dynamics dataset

**DOI:** 10.1016/j.dib.2021.107094

**Published:** 2021-05-06

**Authors:** Arjen Aykan Kılıç, Metehan Yıldırım, Emin Anarım

**Affiliations:** a Boğaziçi University Computer Engineering; b Boğaziçi University Electrical and Electronics Engineering

**Keywords:** Mouse dynamics, Authentication, Verification, Insider threat, Behavioral biometrics, Remote unauthorised access

## Abstract

Mouse dynamics is a hot topic under study in session-based authentication and intrusion detection in privileged access management systems. We contribute to this topic, which develops with the increase of big data capabilities, with a missing data set in the field. This data article describes an extensive dataset with free usage mouse dynamics data. This dataset has seven variables and 24 users with 2550 h of active usage data. Each user has training data, internal attack test data, and external attack test data. An application in Python programming language that continuously listens to mouse movements and mouse clicks and writes them into a file with a timestamp, foremost window name, and mouse action details, is implemented. Among 24 unique users, we labeled five users with the least amount of data as external users since their data would be weak for training purposes, and we can test external threat detection. Each user has training data consisting of 10 days with the most frequent mouse usage, and reminder days of data are used for internal attack test data. This dataset is highly suitable for testing the under-development procedures against the insider threat, remote unauthorised access, and physical access.

## Specifications Table

**Table:** 

Subject	Computer Science, Information Technologies, Cryptography and Cybersecurity
Specific subject area	Mouse Usage Behavior
Type of data	Table
How data were acquired	We implemented a software program and installed them on the users’ computers to acquire the data.
Data format	Raw Anonymized
Parameters for data collection	Dataset is collected from 24 participants who are using an external mouse. All participants were older than 18 years old.
Description of data collection	Users were installed on the work computers provided by the software company with the mouse movement collection software developed by us and general usage data was collected for 1 month.
Data source location	Boğaziçi University, Istanbul, Turkey
Data accessibility	Direct URL to data: https://data.mendeley.com/datasets/w6cxr8yc7p/2

## Value of the Data

• This dataset includes mouse usage behavior patterns of 24 different users. Differently from similar datasets, it has an excessive amount of sessions. Also, it includes more columns that can be used to infer more accurate results.
• Dataset can be valuable for students, engineers, and especially researchers studying behavioral biometrics. Similar datasets have small amounts of data with a low number of users, where we have 24 unique users with 157.377 different sessions.
• This dataset can be used to analyze mouse usage patterns, train neural networks and deep learning models for user identification and verification.

## 1 Data Description

This dataset includes mouse behavior data which is collected from 24 different participants. Dataset is split into three categories; training, external test, and internal test, with 157.377 sessions in total. These categories are explained in detail in the next chapter. In order to measure classification performance, we have to know which test sessions really belongs to the corresponding user and which test sessions do not. We created a file in the dataset directory named “labels.csv” for this purpose, which has two columns; session name and authenticity of the session. Session names are unique. If a test session belongs to the corresponding user, we label it 0 (authorized). If that session belongs to another user, we label it 1 (unauthorised). Each user has its own directory for session files. There are three directories for training data, external test data, and internal test data in each users' directory, which include session files.

Collected data has 7 columns; action type, timestamp, mouse cursor X location, mouse cursor Y location, clicked button (None if it is not a click), click state (Move, Pressed or Released) and foremost application window name respectively. Dataset columns are listed with detailed descriptions in Table 1.

**Table 1:** Detailed Column Descriptions of Dataset.

Variable	Type	Description
Action Type	Categorical	Movement or click
Timestamp	Numeric	Floating point number expressed in seconds since the epoch in UTC
X	Numeric	X location of the mouse cursor
Y	Numeric	Y location of the mouse cursor
Button	Categorical	Indicates which button is clicked (Left or Right). None if it is a movement.
State	Categorical	Button state for clicks (Press or Release). Move if it is a movement.
Window Name	Categorical	Foremost application window name

For all users, browsing (Google Chrome, Mozilla Firefox, Safari, etc.) is the most time spent action by far with 51 percent. Development applications (VMWare, Docker, PyCharm, etc.) are second with 19 percent, Office applications (MS Word, MS Excel, LibreOffice, etc.) are third with 17 percent, filesystem applications (System Preferences, OS programs, setup files, etc.) are fourth by 11 percent, and finally, entertainment applications (Spotify, Discord, video games, etc.) are fifth by 2 percent.

## 2 Experimental Design, Materials and Methods

Experimental users were selected from different positions from a software company, allowing users to use different programs and tools in the office environment. Each user participating in the experiment is loaded with a program that is launched at startup. This program is designed to collect the user's mouse movements without being tied to a specific task. This program is based on the listener callbacks are invoked directly from an operating thread on OS platforms. Thus, users are not prevented from performing daily activities since very little processing power, and memory are needed while collecting mouse movements. Each user uses this program on their computer for one month, and the mouse movements are recorded into .csv files. The raw data records are encrypted with the user's password and taken to the platform where the preprocessing is performed securely.

The raw data records contain the recording time, x and y coordinates, button, state, and the process name of the window that is open while the motion is executed. The first preprocessing is done to divide the process names of the users into the activity type. We have collected all the process names in 5 groups: file system, browsing, development, office, entertainment. This is achieved by manually tagging all process names in an Excel file. Operating system-induced noises and incomprehensible process names in process names were collected under the file system label. We have determined that incomprehensible process names are related to the library we are using, having problems catching “explorer.exe” in the Windows operating system.

After the raw data is tagged, it is divided into sessions. To ensure that training and test data are separate for all experiments, we decided to separate training and test data to be collected in separate folders. Overall, each user has their own training sessions, “internal_threat” sessions in which performance against internal threats is measured, and “external_threat” sessions where performance against external threats is measured.

Preparation of training sessions starts with selecting the target user's ten most active days. These active days are the days with the most data pertaining to the sessions. The data of each day are shared in the training folder as separate sessions. Thus, 10 training sessions for each user are obtained. Before dividing the sessions into folders to be created for performance measurement against internal and external threats, sessions are created with 30 to 100 actions selected from each user's remaining days. Action description is essential here. We split the behavior of users into five main actions. These are free mouse movement (MM), drag and drop (DD), left single click (LC), right single click (RC), double click (DC). Rules are defined to interpret lower-level events into these higher-level actions.

Although this data set is offered as benchmark data for detecting unauthorised account usages, during its creation, no such misuses were carried out. To measure performance against internal and external attacks, the test sessions belonging to the target user and some of the test sessions of other users are randomly selected and collected in the same folder. Thus, the attacks are imitated. Here, all sessions in the “internal_test” folder are selected from the test sessions of the users in the training folder. However, from the sessions in the “external_test” folder, those representing unauthorised sessions are selected from users who are not in the training set. We make the distinction here by selecting 19 users with the most active data from 24 users' data pool for training sessions. We convert all data of the remaining five users with relatively less data into test sessions and distribute them to the “external_test” folder. In conclusion, we have 19 users' data for training, 19 users' data for internal testing, and 5 users' data for external testing.

## Ethics Statement

**Fig. 1:**
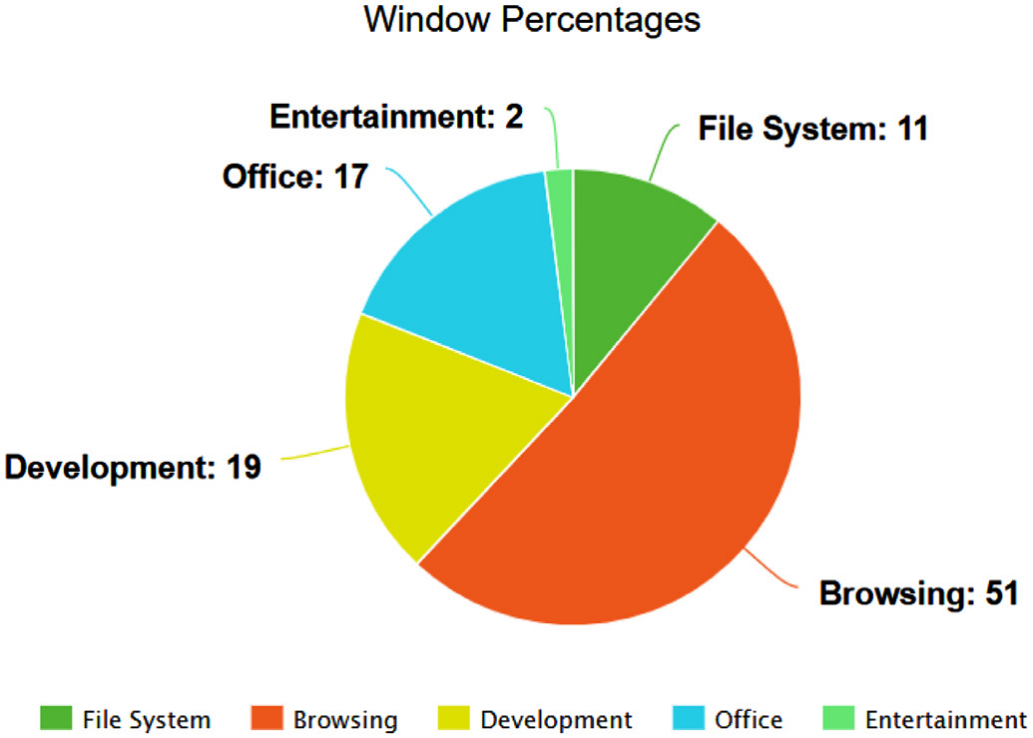
Foremost Window Category Percentages While Using Mouse.

**Fig. 2:**
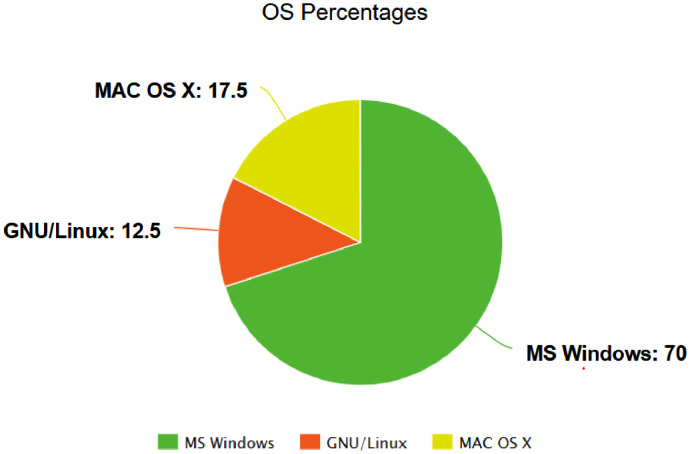
Operating System Usage of Participants.

The authors declare that they have followed the general ethics rules of scientific research performance and publishing. Participants were volunteers and they were informed. In addition, data is anonymized in terms of process names and usernames. (Figs 1 and 2).

## CRediT Author Statement

**Arjen Aykan Kılıç:** Software, Data curation, Visualization; **Metehan Yıldırım:** Data gathering, Methodology, Conceptualization; **Emin Anarım:** Supervision.

## Declaration of Competing Interest

The authors declare that they have no known competing for financial interests or personal relationships that could have appeared to influence the work reported in this paper.

